# Minimally-invasive re-stabilisation of loosened hip implants using percutaneous periprosthetic cement injections: a feasible option to reduce pain in frail patients

**DOI:** 10.1177/11207000251415370

**Published:** 2026-03-18

**Authors:** Kiki Q de Smidt, Shaho Hasan, Perla J Marang-van de Mheen, Demien Broekhuis, Rob GHH Nelissen

**Affiliations:** 1 Department of Orthopaedics, Leiden University Medical Centre, Leiden, the Netherlands; 2 Safety and Security Science, Faculty of Technology, Policy and Management, Delft University of Technology, Delft, the Netherlands

**Keywords:** Loosening, total hip arthroplasty, cement injection, percutaneous, minimal invasive, frail

## Abstract

**Introduction:**

Loosening of hip implants causes severe pain and disability. Revision surgery, the standard treatment, carries high risks, especially for frail patients. Therefore, a less-invasive treatment option is needed for patients with incapacitating pain. We evaluated the safety and efficacy of percutaneous periprosthetic cement injection (PPCI) to restabilise loosened hip implants.

**Methods:**

All patients treated with PPCIs of hip implants between 2004 and 2020 were retrospectively included (median follow-up 24 months, IQR 11–51). Cement was injected in the periprosthetic space of the loosened hip implant under fluoroscopy. Patient characteristics and the Charlson Comorbidity Index (CCI) were measured. Pain reduction was measured by the Harris Hip pain-subscore and adverse events were recorded.

**Results:**

34 patients were included (mean age 83 years, median CCI 6 points). 10 patients had a second PPCI after 12 months (median, IQR 7–27). 2 patients needed revision surgery. 30 patients had pain reduction and 4 had no pain reduction. Adverse events included femoral fissure during needle placement (*n* = 1) and progressive migration of a loosened acetabular cup (despite the cement injection; *n* = 2).

**Conclusions:**

PPCIs for loosened hip implants is feasible in frail patients with pain reduction in the majority of patients and has limited adverse events.

## Introduction

Aseptic loosening of total hip arthroplasties (THAs) typically causes severe pain and loss of independence, leading to a reduced quality of life, particularly in elderly patients.^1,2^ The preferred treatment is revision surgery, but it is demanding, carries substantial blood loss, and increased the risk of infection and other complications.^1^ Additionally, many patients may experience worse outcomes after revision surgery, and in elderly patients with significant comorbidities, the procedure carries higher risk of morbidity and mortality.^3–5^ Therefore, a less invasive treatment would be preferable for these patients.

Several methods to prevent loosening have recently been explored, including bisphosphonates and a single infusion of zoledronic acid.^6,7^ However, alternatives for treating aseptic loosening remain limited. Re-stabilisation of the loosened implant using percutaneous periprosthetic cement injection (PPCI) is an experimental option, used in patients for whom revision surgery is not possible due to severe comorbidities or refusal of patients.^1,8^ De Poorter et al.^1^ first assessed this technique alongside gene therapy aimed at destroying the periprosthetic loosening membrane. A phase 1 dose escalation trial demonstrated that this combination was a feasible treatment for elderly patients unable or not willing to undergo revision surgery.^1,9,10^ However, the short- and midterm clinical outcomes of PPCI without gene therapy have not been assessed. Therefore, this study evaluated the safety and the efficacy of minimal invasive re-stabilisation of loosened hip implants using PPCI with or without gene therapy in frail patients.

## Methods

### Design and patients

**Figure 1.**
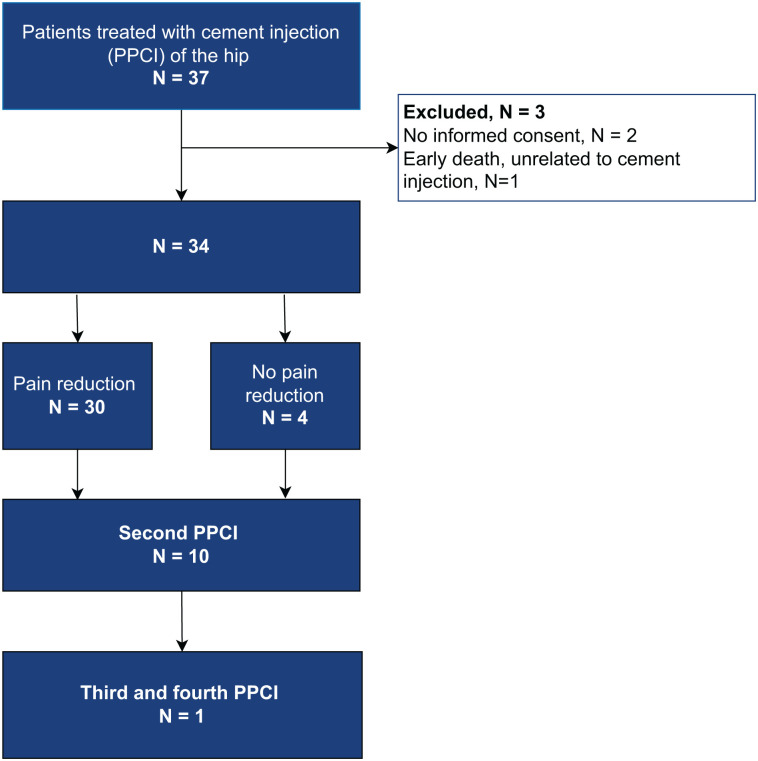
Inclusion flowchart.

We describe the outcomes of a novel treatment for frail patients with loosened hip implants who underwent minimally invasive re-stabilisation using PPCI at the Leiden University Medical Centre between 2004 and 2020. All patients experienced pain and/or severely limitations in mobility and daily function. Loosening (acetabular and/or femoral) were diagnosed based on patient-reported pain and radiographic evidence, including periprosthetic radiolucent lines or implant migration. Eligible patients were those considered frail, for whom revision surgery posed high risks. Eligibility was determined by an orthopaedic surgeon (RN), with most referrals coming from other hospitals. Frailty was assessed preoperatively using the Charlson Comorbidity Index (CCI), a validated predictor for 10-year mortality.^11,12^ Patients with pelvic or hip tumours (*n* = 2) and those without pre- and postoperative pain assessment (*n* = 4) were excluded. This resulted in 34 included patients (Figure 1), 11 of whom participated in Phase I trial of dose-escalating gene therapy followed by PPCI.^1,9^ The patients initially included in the phase I trial were treated with virus-directed enzyme prodrug therapy (VDEPT) followed by percutaneous prosthesis cement injection (PPCI). As the first patients treated with PPCI showed promising results, particularly in this frail population, the use of PPCI was continued in selected patients in the following years.^1,9^ None of the patients included in this study had a documented history of periprosthetic joint infection (PJI).

Patients could undergo a second (or more) PPCI procedure if they experienced persistent or recurrent symptoms and if periprosthetic radiolucent lines were visible on radiographs.

### Procedure

PPCI eligibility was determined through clinical examination (e.g., inability to lift a straight leg, pain hip movement), radiographs (periprosthetic radiolucencies or implant migration), and CT imaging to locate the largest periprosthetic space for cement injection. All PPCI procedures were performed in the operating room under sterile conditions under anesthesia. During the procedure, these areas were identified via CT or fluoroscopy. 3–5 needles (Zimmer Biomet, 3.2 × 100 mm, Warsaw, IN, USA or Jamshidi, 14–16 Gauge, Baxter Healthcare Corporation, Deerfield, IL, USA) were inserted, and polymethylmethacrylate bone cement (PMMA) (Zimmer Biomet, later replaced by SpinePlex, Stryker, Warsaw, IN, USA) was injected into the periprosthetic spaces. Cement flow was monitored fluoroscopically, with injection halted if leakage towards soft tissue or the hip joint was observed to minimise potential cytotoxic and thermal damage to soft tissue. For patients with persistent pain, additional PPCIs were performed if radiolucent periprosthetic spaces remained visible on radiographs.

### Outcome measures

Efficacy was assessed using the pain subscore of the Harris Hip Score (HHS), recorded preoperatively and at follow-up visits. HHS pain scores were classified into 6 levels: totally disabled, crippled, pain in bed (0 points); marked pain, serious limitation of activities (10 points); moderate pain, tolerable but makes concessions to pain (20 points); mild pain, no effect on average activities (30 points); slight, occasional pain (40 points); or no pain, ignores pain (44 points).^13^ Missing HHS pain scores were supplemented using electronic health records, pain visual analogue scale (VAS) scores or the Hip disability and Osteoarthritis Outcome Score (HOOS) pain subscores, independently reviewed by 2 researchers (KS, SH) with discrepancies resolved by consensus. Pain reduction was defined as an increase of ⩾6 (i.e., ⩾2SD) HHS points on the pain subscore between preoperative and the highest postoperative score.^14,15^

Adverse events occurring during the procedure or follow-up were recorded and classified as cement injection-related or unrelated, based on review by researchers (KS, SH) and an orthopaedic surgeon (RN). Some systemic adverse effects were attributed to gene therapy.^1,9^

### Statistics

Means and standard deviations (SD) or 95% confidence intervals (95% CI) were used for normally distributed descriptive variables. Median and interquartile ranges (IQR) or ranges were used for skewed data. SPSS (IBM SPSS Statistics 25.0; IBM Corp, Armonk, NY, USA) was used for the analyses. A *p*-value < 0.5 was considered statistically significant.

### Ethics

Approval for the present study was waived by the Medical Research Ethics Committee (MREC) Leiden-Den Haag-Delft (G20.151) and informed consent was acquired from all patients prior to inclusion.

## Results

34 patients were included (Table 1), with a mean age of 83 years (SD 5.0); 26 (76%) were female. The mean body mass index (BMI) was 25 kg/m^2^ (SD 3.5). The median Charlson Comorbidity Index (CCI) was 6 points (IQR 5–7), corresponding to a median estimated 10-year survival of 2% (IQR 0–21). The mean time from primary hip surgery to cement injection was 17 years (SD 6.7). 11 patients had prior gene therapy.^1^ The median follow-up after PPCI was 24 months (IQR 11–51). During follow-up, 25 patients died (median 4 years after PPCI, IQR 2.5–6.5), at a mean age of 88 years (SD 4.9).

**Table 1.** Patient characteristics total.

Baseline variable	Total *n* = 34	Gene therapy *n* = 11	Without gene therapy *n* = 23
*Age at first PPCI in years, mean (SD)*	83 (5.0)	81 (5.4)	84 (4.7)
*Gender, n*			
— Female	26	11	15
Alive at final follow-up, n	8	1	7
BMI, mean (SD)	25 (3.5)	25 (3.3)	26 (3.6)
Gene therapy, n	11	11	0
Diagnosis prior to primary arthroplasty, n			
— Primary osteoarthritis	13	7	6
— Secondary osteoarthritis	8	3	5
— Missing	13	1	12
CCI – points, median (IQR)	6 (5–7)	6 (5–7)	5 (5–6)
CCI – estimated 10-year survival (%), median (IQR)	2 (0–21)	2 (0–21)	21 (2–21)
Time between primary surgery and first ppci in years, mean (SD)	17 (6.7)	19 (8.5)	17 (5.9)
Type of implant, n			
— Total hip arthroplasty	33	10	23
— Hemiarthroplasty	1	1	0
Cemented implant, n	30	11	19
Location cement injection, n			
— Femur	17	5	12
— Acetabulum	2	0	2
— Femur and acetabulum	15	6	9
Follow-up in months, median (IQR)	24 (11.0–50.8)	24 (6.0–38.0)	24 (12.0–60.0)

PPCI, percutaneous periprosthetic cement injection; SD, standard deviation; BMI, body mass index; CCI, Charlson Comorbidity Index; IQR, interquartile range.

### Primary outcome

The median preoperative HHS pain-subscore was 10 points (‘marked pain’). Postoperatively, 30 patients (88%) experienced pain relief (median HHS pain increase: 32, IQR 20–34), while 4 had no improvement. 2 of these had unresolved cup loosening. No difference in pain reduction was found between those with or without gene therapy (Table 2). 10 patients underwent a second PPCI for recurrent or persistent pain after a period of pain relief (mean 12 months, IQR 6.5–26.8). or persistent pain. The median time between the first and second PPCI was 12 months (IQR 6.5–26.8) (Table 2). 1 patient required a third and fourth PPCI. 2 patients underwent revision surgery for persisting pain due to progressive loosening or periprosthetic fracture (Table 2), described in more detail below.

**Table 2.** Primary outcome and adverse events related to PPCI in patients with and without interface removal (i.e., gene therapy).

Outcome measure	Total *n* = 34	Gene therapy *n* = 11	Without gene therapy *n* = 23
*Pain reduction during follow-up*			
— Yes	30	8	22
— No	4	3	1
*Repeated PPCI after first PPCI, n*			
— 1 (2^nd^ PPCI)	10	5	5
— 2 (3^rd^ PPCI)	1	1	0
— 3 (4^th^ PPCI)	1	1	0
Time between 1^st^ and 2^nd^ PPCI in months, median (IQR)	12 (6.5–26.8)	10 (8.5–30.5)	13 (1.5–24.5)
adverse events related to the cement injection, n^a^			
— Fracture after ambulation	2	1	1
— Fissure during PPCI	1	0	1
— greater trochanteric pain	4	1	3
— Quadriceps haematoma	2	1	1
— Progressive migration of the cup	2	1	1
— Patients with adverse events related to the PPCI, n	9	4	5
Revision surgery of THA, n	2	1	1

PPCI, percutaneous periprosthetic cement injection; IQR, interquartile range; THA, total hip arthroplasty.

Pain reduction if increase of >6 points on the HHS-pain subscore.

a Patients could have >1 adverse event.

### Adverse events

**Figure 2.**
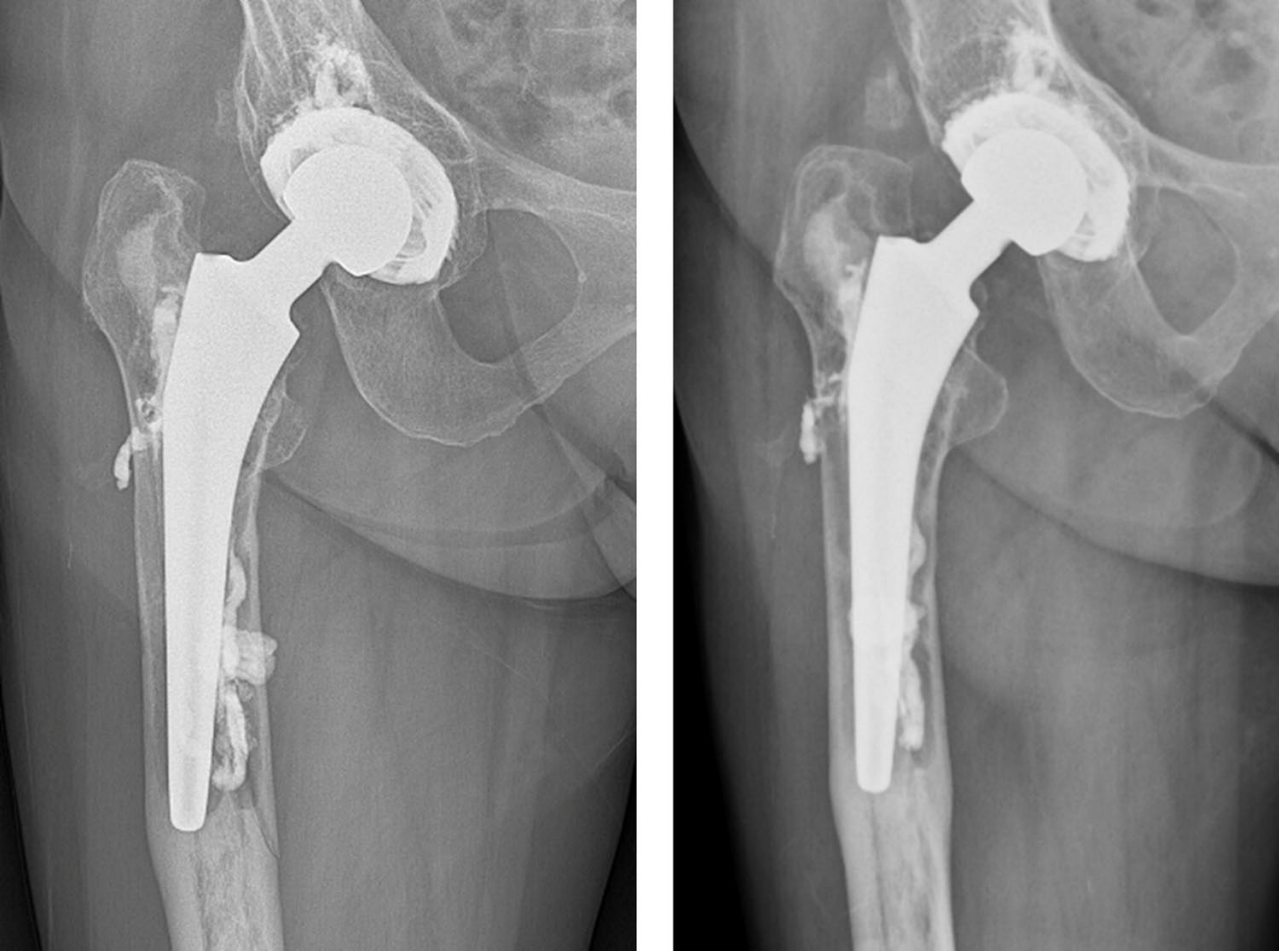
X-ray of a patient’s right hip after cement injection, showing a non-dislocated fissure, which occurred during needle positioning (left radiograph). The fissure was fully consolidated within 6 months after the PPCI (right radiograph).

**Figure 3.**
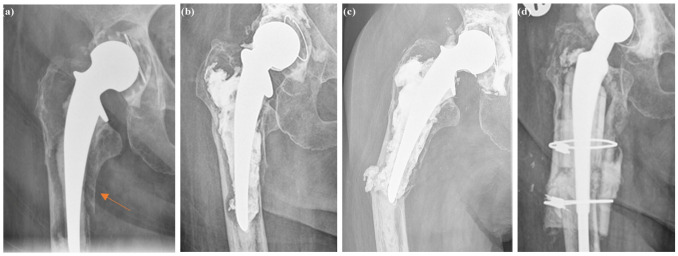
Radiographs of a patient’s right hip before the first PPCI with a fissure of the femoral bone in Gruen Zone 5 (a), after the first PPCI (b), 4 weeks after the second PPCI (c), and after extensive revision surgery (d), showing the course of the fracture.

Most patients (85%) were discharged the same day; the rest left the next morning. 9 patients experienced PPCI-related adverse events (Table 2). 1 had a femoral fissure during needle positioning, which healed uneventful within 6 months (Figure 2). In a second patient with gross implant loosening, a femoral fissure (Gruen zone 5) was present before cement injection (Figure 3). She was 76 years old, and wheelchair bound. Despite the periprosthetic fissure and the loosened hip stem, she only opted for PPCI, which resulted in pain relief. But after partial weight-bearing for 6 weeks, she sustained a periprosthetic fracture, which needed revision surgery (Figure 3). 2 patients developed a painful quadriceps haematoma after cement injection, which resolved spontaneously. 4 patients developed greater trochanteric pain which resolved after local corticosteroid injection. In the gene therapy group, systemic adverse events occurred related to the gene therapy.^9,10^ 2 patients with preoperative severely loosened acetabular cups had progressive migration of the cup despite the cement injection (Table 2). 1 American Society of Anesthesiologists (ASA) III patient with rheumatoid arthritis reported no pain relief during follow-up. Another ASA IV patient, previously treated with a total hip prosthesis after septic arthritis, underwent PPCI 16 years post-implantation. The initial PPCI led to cup subluxation without pain improvement; a second PPCI targeting the femoral stem was also ineffective. The patient died 6 months later, from causes unrelated to the PPCI.

## Discussion

This study evaluated the safety and efficacy of minimally-invasive percutaneous re-stabilisation of loosened hip implants in frail patients using percutaneous cement injections in the periprosthetic interface. Pain was reduced in 88%, though 10 required additional PPCIs. However, 4 patients experienced no pain reduction, including 2 who underwent PPCI for a loosened cup.

**Figure 4.**
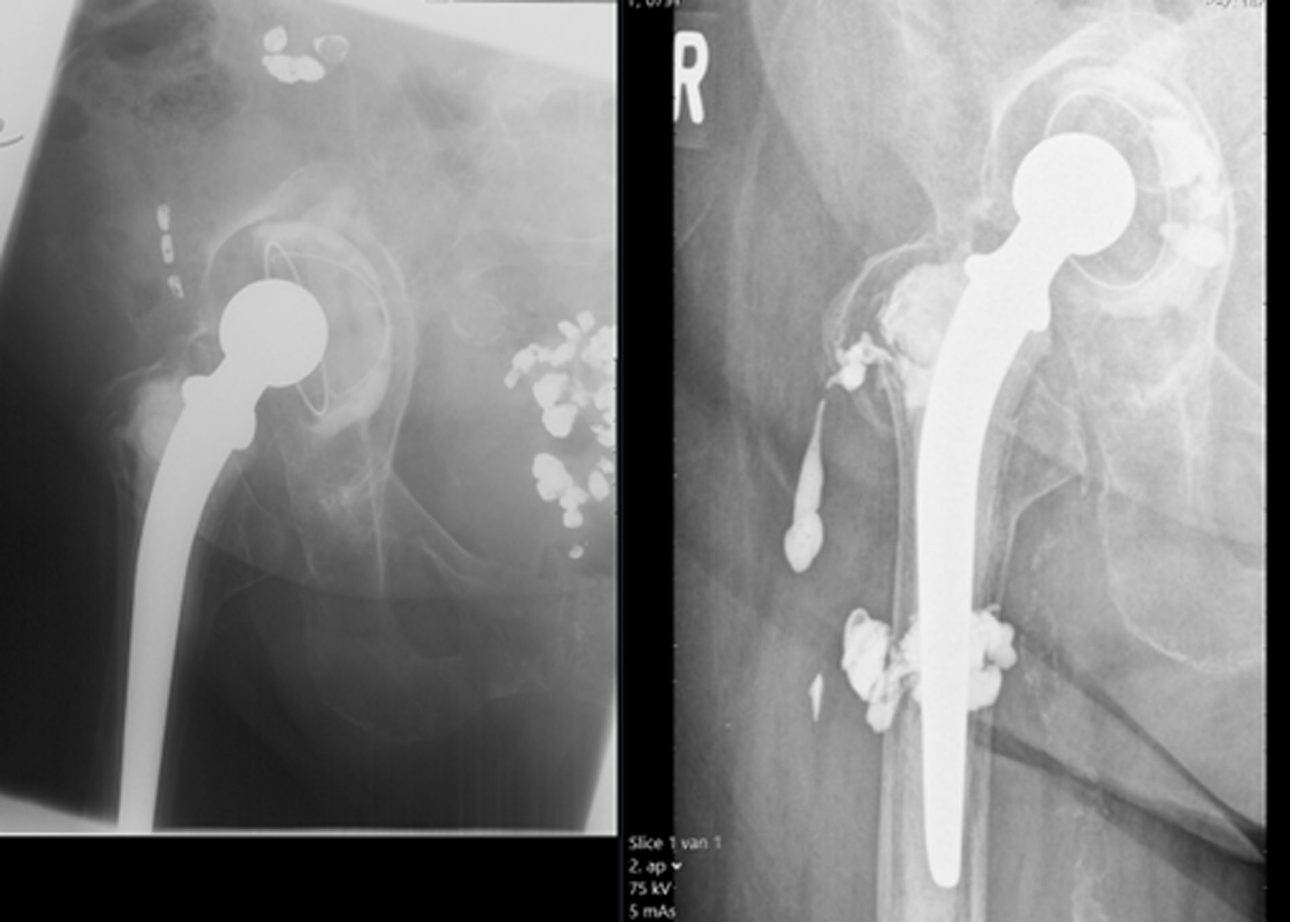
PCCI of the femur and acetabulum showing preoperative subluxation of the cup with further dislocation of the cup postoperatively.

Our technique is ineffective for severely loosened acetabular components with gross cup migration (*n* = 2) (Figure 4), as PPCI cannot prevent further displacement seen on successive pre-PPCI radiographs. As a result, PPCI is no longer used for such cases. Despite being a percutaneous technique, some adverse events directly and indirectly related to this technique occurred, but overall, PPCI is a safe technique and could be a viable option to treat symptoms of loosened hip implants in frail patients for whom revision surgery would have high risks. However, patients with osteopenic periprosthetic bone, experiencing pain relief, may increase mobility which raises the risk of periprosthetic fractures. Since bone remodelling takes months, walking aids remain essential in the early postoperative period, as demonstrated by patients who sustained fractures after tripping at home.

Our percutaneous periprosthetic cement injection technique is a palliative option for patients with painful loosened orthopaedic implants, currently available only at our tertiary referral centre. A previous Phase I-II study using gene-directed enzyme prodrug therapy (GDEPT) with cement injection showed promising clinical results.^8–10^ However, due to the complexity of GDEPT, alternative methods to remove interface tissue such as laser or waterjet, have been explored.^16–18^ A promising innovative approach involves stimulating synovial pluripotent cells of the interface tissue to form bone, which has shown success in *ex-vivo* studies.^19,20^

Supporting the removal of interface tissue before cement filling, Malan et al.^1,8^ compared radiographs before and after PPCI in patients with and without GDEPT-based interface removal (i.e., cell apoptosis and rinsing with ethylenediamine tetraacetic acid (EDTA)). They found that eliminating the periprosthetic interface tissue resulted in better bone-cement filling.^1,8,17,21^ However, the present study suggests that even without interface removal, patients experienced pain reduction comparable to those who underwent GDEPT before PPCI.

This study has some limitations. First, periprosthetic filling was restricted by extra-osseus cement leakage or leakage into the hip joint when cement was injected under fluoroscopy. This was a key reason why some patients required multiple PPCIs. Additionally, while PPCI improves implant stability, it is less effective than revision surgery, where interface tissue is removed, and the stem is optimally pressurised in cement. Second, follow-up visits were limited in some cases due to patients’ advanced age, mobility issues, and reliance on carers for transport, leading to variability in follow-up periods and outcome comparisons. However, given the mean age of 83 years – close to the Dutch life expectancy of 80 for men and 83 for women – pain reduction with minimal risk remains the priority, even if temporary. Also, the follow-up period was relatively short which restricts conclusions about long-term outcomes. Third, as a tertiary centre, our patient group likely represents a more severe subset with incapacitating pain and advanced loosening, limiting the generalisability to those with milder symptoms. In the future, early implant loosening may be managed by transforming interface into bone, potentially serving as a maintenance procedure to delay or prevent extensive revision surgery.^21^ Fourth, this study was not powered for functional outcomes such as the HHS, and the high mortality rate reflects the frailty of the cohort, limiting conclusions but we support further investigation of PPCI as a palliative option in symptomatic loosened hip implants. Also, radiographic outcomes were not reported as the primary focus was on clinical outcomes. Last, a subset of the patients needed a repeat PPCI and some patients had no benefit from PPCI, reflecting the palliative nature of the procedure and underscoring the importance of careful patient selection.

In conclusion, 88% of patients treated with PPCI’s experienced pain reduction with minimal adverse events, even after multiple procedures. This study supports the feasibility of minimally invasive re-stabilisation of loosened hip stems in frail patients. However, caution is advised for grossly loosened, migrating cups. PPCI may also be considered for other loosened orthopaedic implants in frail patients, provided the benefit-risk ratio is carefully assessed.
